# Efficacy of Immune Checkpoint Inhibitor Monotherapy for Advanced Non-Small-Cell Lung Cancer with *ALK* Rearrangement

**DOI:** 10.3390/ijms21072623

**Published:** 2020-04-09

**Authors:** Yuko Oya, Hiroaki Kuroda, Takeo Nakada, Yusuke Takahashi, Noriaki Sakakura, Toyoaki Hida

**Affiliations:** 1 Department of Thoracic Oncology, Aichi Cancer Center Hospital, 1-1 Kanokoden, Chikusa-ku, Nagoya, Aichi 464-8681, Japan; yshima@aichi-cc.jp (Y.O.); 107974@aichi-cc.jp (T.H.); 2 Department of Thoracic Surgery, Aichi Cancer Center Hospital, 1-1 Kanokoden, Chikusa-ku, Nagoya, Aichi 464-8681, Japan; t.nakada@aichi-cc.jp (T.N.); y.takahashi@aichi-cc.jp (Y.T.); nsakakura@aichi-cc.jp (N.S.)

**Keywords:** *ALK*, *EGFR*, non-small-cell lung cancer (NSCLC), immune checkpoint inhibitor (ICI), adenocarcinoma

## Abstract

Programmed death-ligand 1 (PD-L1) expression is a predictor of immune checkpoint inhibitor (ICI) treatment efficacy. The clinical efficacy of ICIs for non-small-cell lung cancer (NSCLC) patients harboring major mutations, such as *EGFR* or *ALK* mutations, is limited. We genotyped 190 patients with advanced lung adenocarcinomas who received nivolumab or pembrolizumab monotherapy, and examined the efficacy in NSCLC patients with or without major mutations. Among the patients enrolled in the genotyping study, 47 patients harbored *EGFR* mutations, 25 patients had *KRAS* mutations, 5 patients had a *HER2* mutation, 6 patients had a *BRAF* mutation, and 7 patients had *ALK* rearrangement. The status of PD-L1 expression was evaluated in 151 patients, and the rate of high PD-L1 expression (≥50%) was significantly higher in patients with *ALK* mutations. The progression-free survival was 0.6 (95% CI: 0.2–2.1) months for *ALK*-positive patients and 1.8 (95% CI: 1.2–2.1) months for *EGFR*-positive patients. All patients with *ALK* rearrangement showed disease progression within three months from the initiation of anti-PD-1 treatment. Our data suggested that ICI treatment was significantly less efficacious in patients with *ALK* rearrangement than in patients with *EGFR* mutations, and PD-L1 expression was not a critical biomarker for ICI treatment for patients with one of these mutations.

## 1. Introduction

Anaplastic lymphoma kinase (*ALK*) is a tyrosine kinase that is constitutively activated in certain types of cancers due to genetic abnormalities, such as chromosomal translocations, gene amplification, and point mutations [1,2]. In 2007, Mano et al. [3] reported a small translocation within chromosome 2 in lung adenocarcinoma cells, which results in the generation of an echinoderm microtubule-associated protein-like 4 (*EML4*)–*ALK* fusion gene. The *ALK* is a receptor tyrosine kinase that is responsible for cell growth, but when it is fused with EML4, the enzyme activity is increased to become an EML4–ALK kinase that has strong oncogenic potential. Additionally, Mano et al. [4] reported the oncogenicity of the *EML4–ALK* fusion gene in genetically modified mice, which produced *EML4–ALK* kinase specific to lungs and developed multiple lung cancers soon after birth.

*ALK* gene rearrangement occurs in a small portion of patients with non-small-cell lung cancer (NSCLC) [5], but it accounts for about 30%–40% of lung adenocarcinoma in young individuals, and is present in many non-smokers and patients with epidermal growth factor receptor (*EGFR*)-mutated lung cancer [6,7,8]. *ALK* tyrosine kinase inhibitors (TKIs) are effective in NSCLC patients with *ALK* gene rearrangement. Crizotinib, a first-generation *ALK* inhibitor that was originally developed as a *c-Met* inhibitor, also potently inhibits *ALK* kinase [9,10]. Crizotinib showed clinical benefit for NSCLC patients with *ALK* rearrangement beyond cytotoxic chemotherapy in the PROFILE 1007 trial and PROFILE 1014 trial, and has been approved and used in clinical practice [9,10]. However, after the initial positive response, all patients developed resistance to crizotinib after approximately one year. The most frequent resistance mechanism is the L1196M gatekeeper mutation, but resistance mechanisms of *ALK* inhibitors are more diverse than those of *EGFR* TKIs [11,12,13]. Second-generation *ALK* inhibitors have been developed as effective drugs for *ALK*-positive NSCLC patients who have acquired resistance, such as alectinib, which has shown remarkable efficacy after acquired resistance to crizotinib in clinical trials [14]. To answer the question of whether sequential treatment with alectinib after crizotinib is better than alectinib for *ALK*-positive NSCLC as an initial treatment, alectinib and crizotinib were previously compared as a first-line treatment for *ALK*-positive NSCLC. The results showed that alectinib was superior to crizotinib as a first-line treatment for *ALK-*positive NSCLC [15,16]. However, resistance to alectinib eventually developed. Moreover, the resistance mechanism of alectinib has been reported to be more diverse than that of crizotinib [17]. Approximately 30% of resistance to *ALK* inhibitors is due to gene mutations, but the new acquired resistance mechanisms are diverse [18]. Attempting to overcome resistance by focusing on resistant mutated genes is not a common treatment strategy [19]. Therefore, cytotoxic chemotherapy and immune therapy are needed to treat patients who develop resistance to *ALK* inhibitors. Treatment after acquired resistance to *ALK* inhibitors include other *ALK* inhibitors, but an immune checkpoint inhibitor (ICI) + chemotherapy, which is the standard first-line treatment for NSCLC without a driver oncogene, is a candidate for *ALK* inhibitor resistance. However, there is less data on the efficacy of ICIs for *ALK*-rearranged NSCLC. Among the clinical trials for ICI + chemotherapy, those including *ALK*- and *EGFR*-positive cases have reported the use of atezorizumab + bevacizumab + carboplatin + paclitaxel (ABCP) (IMPOWER 150 trial). For *EGFR/ALK*-positive cases, ABCP showed a 70.6% objective response rate (ORR), and ABCP was superior to BCP in progression-free survival (PFS) and overall survival (OS) in a subgroup analysis (PFS—hazard ratio (HR): 0.59, 95% confidence interval (CI): 0.37–0.94; OS—HR: 0.54, 95% CI: 0.29–1.0). However, only 11 *ALK*-positive cases were included in the IMPOWER 150 trial [20]. Similarly, there are a few reports of *ALK*-positive NSCLC with ICI monotherapy [21,22]. Additionally, in many clinical trials, *ALK* and *EGFR* are administered in combination. Although the backgrounds of patients who have *EGFR*- and *ALK*-positive tumors commonly include non-smokers and young patients [6,7,8], it is unclear whether a similar clinical course is followed in immunotherapy. It has been reported that programmed death-ligand 1 (PD-L1) expression is high in *ALK*-positive lung cancer [23,24]. PD-L1 expression is one of the predictors of efficacy for ICI treatment [25,26]. However, there are a few reports of studies that have investigated the efficacy of PD-1 inhibitors in *ALK-*rearranged NSCLC patients [21,22]. Therefore, we compared the efficacies of ICIs for the treatment of lung adenocarcinoma with *ALK* rearrangement and other oncogene drivers, including *EGFR* mutations.

## 2. Results

### 2.1. Patients and Treatment 

The median follow-up was 10.2 months (range: 0–51 months). The patient characteristics are summarized in Table 1. The median age of the patients was 66 years (range: 32–87 years). All patients had adenocarcinoma, and 141 patients were smokers. The Eastern Cooperative Oncology Group Performance Status (PS) was 0 to 1 for 166 patients and 2 to 3 for 24 patients. Anti-PD-1 therapy was the first-line treatment for 27 patients, the second-line treatment for 70 patients, and the third-line treatment for 93 patients. Nivolumab was used to treat 138 patients, and pembrolizumab was used to treat 52 patients. Forty-seven patients (24%) harbored *EGFR* mutations, 25 patients (13%) had v-Ki-ras2 Kirsten rat sarcoma viral oncogene homolog (*KRAS*) mutations, 5 patients (3%) had a human epidermal growth factor receptor type 2 (*HER2*) mutation, 6 patients (3%) had a B-Raf proto-oncogene, serine/threonine kinase (*BRAF*) mutation, and 7 patients (4%) had *ALK* rearrangement. The status of PD-L1 expression could be evaluated in 117 (62%) patients. Fifty-eight patients had high PD-L1 expression (≥50%), 52 patients had low PD-L1 expression (1%–49%), and 41 patients were PD-L1 negative.

**Figure 1:**
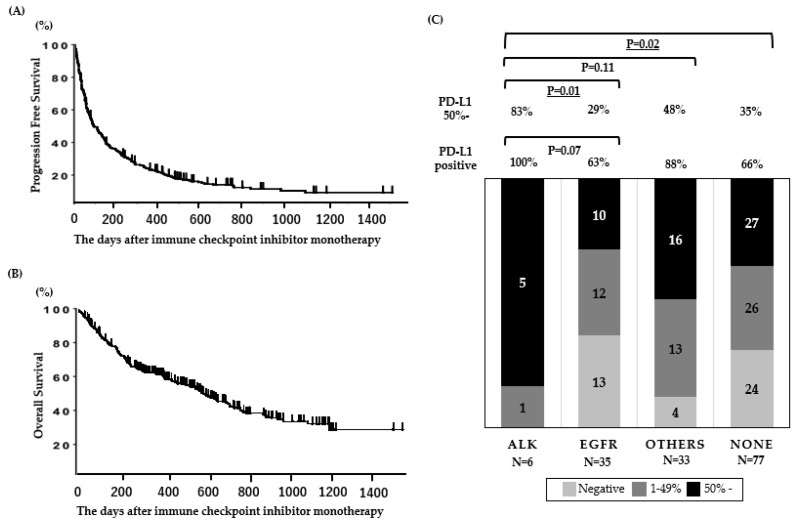
Clinical outcomes and PD-L1 expression in patients treated with ICI. (**A**) Progression-free survival and (**B**) overall survival (N = 190). (**C**) PD-L1 status according to oncogenic subtypes in patients assessed for PD-L1 expression (N = 151). Underlined values indicate a significant difference.

When the database was closed, 23 of the patients were continuing anti-PD-1 treatment. For efficacy measurements, the objective response rate (ORR) and the median progression-free survival (PFS) in all patients (N = 190) were 22% and 2.4 (95% CI: 2.0–3.1) months, respectively (Figure 1A), and the OS of patients treated with anti-PD-1 therapy was 14.3 (95% CI: 10.0–19.3) months (Figure 1B). The PFS and OS data presented in this cohort are comparable to those presented in ICI monotherapy clinical trials [20,25,26,27].

### 2.2. PD-L1 Expression for Each Mutation 

The PD-L1 expression for each mutation is shown in Figure 1C. The rate of high PD-L1 expression (≥50%) was significantly higher in patients with *ALK* mutations than in patients with other mutations, including *EGFR* mutations.

### 2.3. Efficacy of Immune Checkpoint Inhibitors According to Oncogenic Subtype

**Figure 2:**
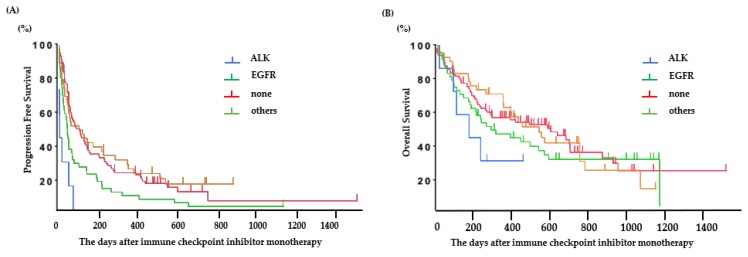
Clinical outcomes in patients treated with ICI. (**A**) Progression-free survival based on each oncogenic subtype and (**B**) overall survival based on each oncogenic subtype.

The PFS was 0.6 (95% CI: 0.2–2.1) months for *ALK*-positive patients, 1.8 (95% CI: 1.2–2.1) months for *EGFR*-positive patients, 3.2 (95% CI: 1.8–7.6) months for patients with other mutations, and 3.2 (95% CI: 2.3–4.8) months for patients without any mutations (Figure 2A). Among patients with *EGFR* or *ALK* mutations, the PFS following ICI treatment was significantly longer in patients with *EGFR* mutations than in those with *ALK* mutations (*ALK*: 0.6 (95% CI: 0.2–2.1) months, *EGFR*: 1.8 (95% CI: 1.2–2.1) months; *p* < 0.01) (Figure 2B). The predictive factors in the univariate and multivariate analyses are presented in Table 2. The good PS (0–1), PD-L1 status (positive), mutation (without *EGFR* or *ALK*), and baseline C-reactive protein (CRP) value (<1.0 mg/dL) were significant factors for PFS of ICI-treated patients.

**Figure 3:**
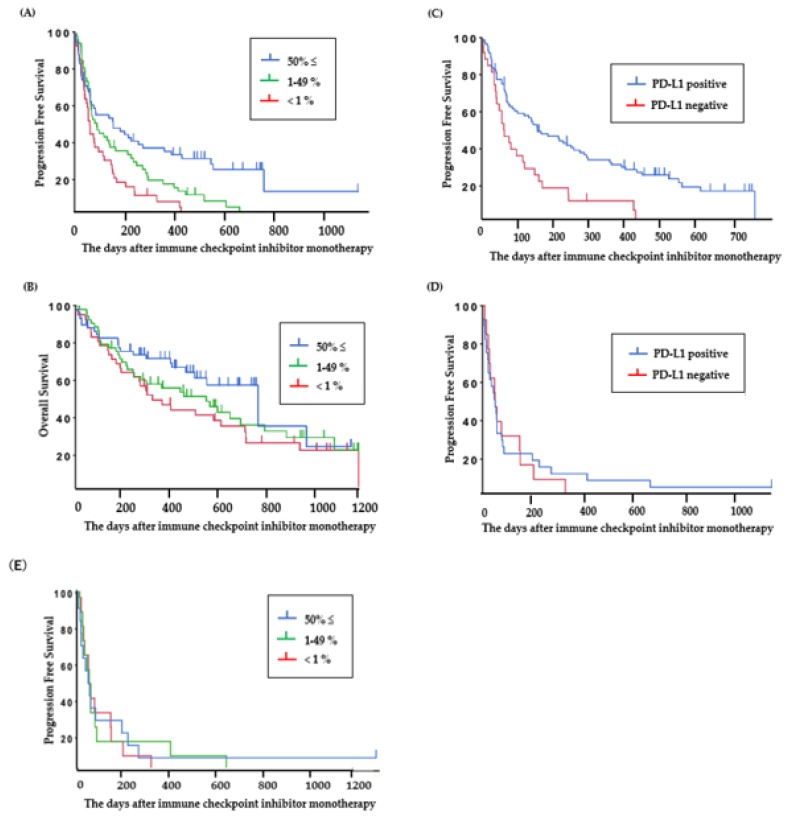
Clinical outcomes in patients treated with ICI. (**A**) Progression-free survival and (**B**) overall survival based on PD-L1 status in all patients viable for PD-L1 assessment (N = 151). (**C**) Progression-free survival based on PD-L1 status in patients without *EGFR* or *ALK* mutations (N = 141) and (**D**,**E**) with *EGFR* or *ALK* mutations (N = 54).

The PFS of ICI-treated patients with PD-L1 high expression (≥50%), PD-L1 low expression (1%–49%), and PD-L1-negative tumors was 5.2 (95% CI: 2.1–9.2) months, 2.9 (95% CI: 2.1–4.8) months, and 2.1 (95% CI: 1.4–3.2) months (Figure 3A), and the OS was 25.4 (95% CI: 15.5–32.0) months, 18.3 (95% CI: 8.3–23.0) months, and 10.9 (95% CI: 6.5–20.3) months, respectively (Figure 3B).

The PFS of ICI was significantly longer in patients who were PD-L1 positive than in those who were PD-L1 negative in the group without *EGFR* or *ALK* mutations (PD-L1 positive: 5.2 (95% CI: 3.1–8.6) months (Figure 3C), PD-L1 negative: 2.1 (95% CI: 1.4–3.8) months; *P* < 0.01), but for patients with *EGFR* or *ALK* mutations, there was no significant difference between the PFS of ICI-treated patients who were PD-L1 positive and those who were PD-L1 negative (PD-L1 positive: 1.8 (95% CI: 1.0–2.1) months, PD-L1 negative: 1.9 (95% CI: 0.9–5.1) months; *P* = 0.83) (Figure 3D). ICI-treated patients with PD-L1 high expression (≥50%) and patients with *ALK* or *EGFR* mutations showed short PFS. The PFS of patients with PD-L1 high expression (≥50%), PD-L1 low expression (1%–49%), and PD-L1-negative tumors in the group with *ALK* or *EGFR* mutations was 1.9 (95% CI: 0.9–5.1) months, 1.9 (95% CI: 0.9–2.7) months, and 1.7 (95% CI: 0.6–2.8) months, respectively (Figure 3E).

All patients with *ALK* rearrangement showed disease progression within three months from the initiation of anti-PD-1 treatment. 

## 3. Discussion

We found that anti-PD-1 therapy was less efficacious in patients with *ALK* rearrangement than in patients with other mutations, including *EGFR* mutations. Recently, several studies that examined the correlation between oncogenic subtype and efficacy of ICIs have been reported [21,22,27,28,29]. Mazieres et al. reported the association between the efficacy of ICI and driver oncogenes and found that patients with actionable mutations (*EGFR*, *ALK*, and *ROS-1*) had poor ICI treatment outcomes [22,30]. The present study is the first to show the difference in ICI efficacy between patients with *EGFR* mutations and *ALK* rearrangement. TKIs are used as first-line treatment for patients with driver oncogenes, but after patients become resistant, ICI treatment is considered at any stage. The efficacy of nivolumab was found to be inferior to that of docetaxel in patients with *EGFR* mutations in the Checkmate 057 trial [27], but there is less data on the efficacies of ICIs for NSCLC with *ALK* rearrangement. Different *ALK* inhibitors are clinically used subsequent to the resistance to *ALK* inhibitors, but ICI + chemotherapy, which is the standard first-line treatment for NSCLC without a driver oncogene, is another option. However, little is known about the efficacy of these regimens in patients with an *EGFR* or *ALK* mutation. The IMPOWER 150 trial [20] reported ICI + chemotherapy efficacy in patients, including those with *ALK* and *EGFR* mutations, and ABCP was used for treatment in the trial. In the *EGFR*-positive cases, ABCP gave a 70.6% ORR, and the HR for BCP therapy was 0.61 (95% CI: 0.29–1.28). However, only 11 *ALK*-positive patients were included in the IMPOWER 150 trial, and the effects of treatment on those patients were not described [20].

In addition, it is not known whether the predictive factors of ICI treatment efficacy are equivalent between patients with and without driver oncogenes. In this study, a multivariate analysis detected that good PS (0–1), PD-L1 status (positive), mutation (without *EGFR* or *ALK*), and baseline C-reactive protein (CRP) value (<1.0 mg/dL) were significant factors for PFS following ICI treatment. There are some reports that ICI treatment has less efficacy in patients with a high CRP level. There are several reports, including our report, that ICI treatment has less efficacy in patients with poor PS or high CRP. It is thought that these are due to immunosuppression and the relationship with inflammatory cytokines [31,32,33], but the reasons are not clear. Therefore, CRP is not a clear biomarker. However, PD-L1 expression, one of the biomarkers of ICI treatment, is important when determining the course of treatment [24,25]. We do not know whether PD-L1 is a suitable biomarker for patients with *EGFR* or *ALK* mutations. In this study, we found that the correlation between PD-L1 expression and the effect of ICI treatment was not strongly associated in patients with *ALK* rearrangement, in spite of the small population included in the study. 

Recently, several studies have shown that oncogenic signals derived from mutations or loss of tumor suppressor genes upregulate the expression of immune checkpoint molecules in cancer cells during immune escape [34]. In *EGFR*-mutated NSCLC, PD-L1 expression was reportedly enhanced, which resulted in the suppression of T-cell function via activation of the PD-1/PD-L1 pathway. Furthermore, it has been reported that the *EML4*-*ALK* rearrangement upregulated PD-L1 expression via HIF-1α and STAT3 in vitro [35]. 

**Figure 4:**
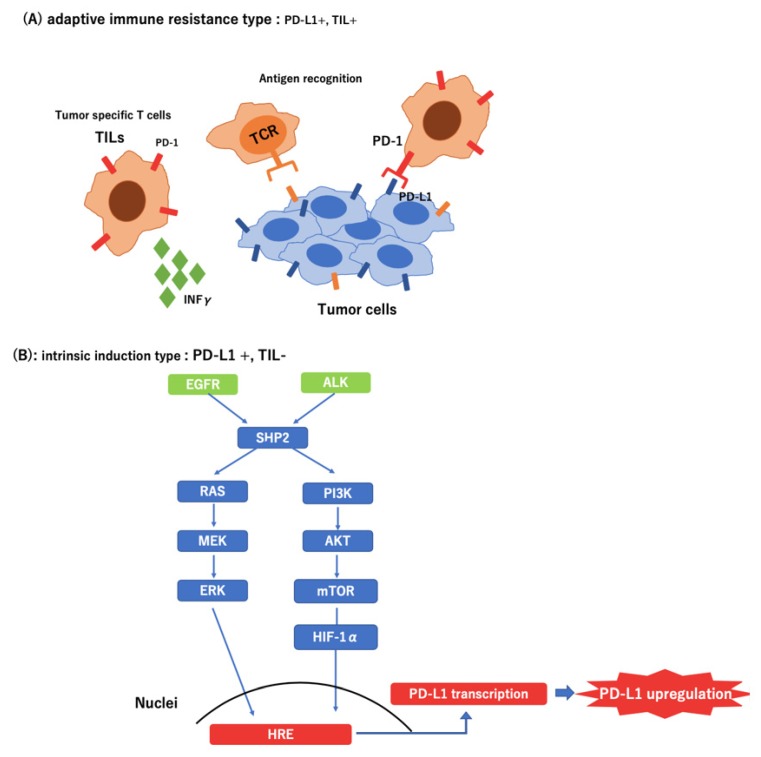
Different types of tumor microenvironment in PD-L1-positive tumors. (**A**) Adaptive immune resistance type: Tumor Infiltrating Lymphocyte (+); (**B**) Intrinsic induction type: Tumor Infiltrating Lymphocyte (−)

The expression of PD-L1 occurs by two mechanisms. First, tumor-infiltrating lymphocytes (TIL) in the tumor microenvironment produce interferon-γ, thereby upregulating PD-L1. Thus, PD-L1 expression may be correlated with the existence of TILs, and PD-L1 may be used as a biomarker of the efficacy of ICI treatment [36,37,38]. Second, PD-L1 is upregulated by a pathway downstream of the driver oncogene [35,39]. In this case, the expression of PD-L1 was increased secondarily with no relationship with the presence of TILs or effects of ICI. In the present study, all patients with *ALK*-positive cases experienced exacerbation within three months after the initiation of ICI treatment, even patients with high expression of PD-L1. This result indicates that PD-L1 expression may not be an effective predictor in patients with lung cancer who have *ALK* or *EGFR* mutations (Figure 4).

Another point to be considered in the treatment with ICI for *ALK*-positive patients is that early treatment with ICI could take away the next treatment opportunity. For example, some patients experience a unique pattern of progression disease called hyperprogression [40,41,42], which is characterized by rapid disease progression after the initiation of ICI. Patients who experience hyperprogression show poor prognosis, and they cannot take the next treatment. Some retrospective studies have also reported an increased risk of hepatotoxicity with the sequential use of ICIs and crizotinib [43]. These data are consistent with the unexpected severe AE rates reported for different combinations of *ALK* inhibitors and PD-1/PD-L1 inhibitors [44]. Patients who show sever AEs cannot take the next treatment until the AEs are reduced. Patients with *EGFR* or *ALK* mutations respond to TKIs dramatically. Even after they acquire resistance to TKIs, they are still subjected to certain cytotoxic chemotherapeutic effects. In particular, pemetrexed has been reported to have good antitumor activity in patients with *ALK* rearrangement [45]. Therefore, it is a great disadvantage that these patients cannot receive the next treatment because an ICI was used earlier. 

In this study, the effect of ICI in patients with other mutations was almost equivalent to the effect in patients without mutations, and PD-L1 expression served as a predictive factor. These differences between *EGFR*, *ALK*, and other oncogenes were not biologically obvious. However, *EGFR* and *ALK* greatly contribute to carcinogenesis as driver oncogenes, and targeted therapy is effective. Other genes, including *KRAS,* may not be as carcinogenic as *EGFR* or *ALK*.

There were several limitations in this study. First, this was a retrospective study at a single institution. Bylicki et al. reported that there were no significantly difference in PFS between the *ALK* group (2.4 months) and the EGFR group (2.2 months). The PFS of *ALK*-positive patients were shorter in our study (0.6 months) compared with the Bylicki study [22]. There are some differences in the Bylicki study and our study, such as race, smoking status, and gender proportion, but these are not sufficient reasons to explain the difference. Therefore, these differences may be accidental due to the small sample size. Therefore, larger studies need to be performed to accurately validate the usefulness of ICI treatment in *ALK*-positive patients. Second, the treatment lines of ICI varied in this study. In lung cancer, it is unknown whether TKIs affect the efficacy of ICI treatment. Treatment with a TKI is recommended for the initial treatment of NSCLC patients with a driver oncogene, and almost all patients with a driver oncogene, except *EGFR* exon 20 insertion, were pre-treated with a TKI in this study. The effects of prior treatment with a TKI may be considered as a reason for poor efficacy of ICI treatment in patients with *EGFR* and *ALK* mutations. Given this limitation, prospective trials will be required to confirm the effect of driver oncogenes on the efficacy of ICI treatments. However, in patients with *EGFR* mutations or *ALK* rearrangement, TKI is firmly positioned as an initial treatment because of its high efficacy, and it is difficult to verify the effect of ICI with or without a treatment history of TKI. A single-arm phase 2 trial for *EGFR*-positive lung cancer in *EGFR* TKI-untreated patients had been initiated, but was stopped during the interim analysis because of poor efficacy [46]. *EGFR* itself may be a factor that lowers the effect of ICI, and the patients’ sex may also be a factor. In addition, in lung cancer patients, pneumonitis frequently occurred in the combination therapy of pembrolizumab and osimertinib in the TATTON trial, and there were concerns about safety in the combination treatment of TKI and ICI [47]. In addition, there have been some reports of pneumonitis caused by treatment with osimertinib after ICI. Therefore, the sequential treatment strategy of TKI followed by ICI is difficult in lung cancer. In addition, it has been reported that the efficacy of ICI will be poor in late-treatment lines. Patients with *EGFR* or *ALK* mutations tend to receive ICI as a late-line treatment because other candidates are preferred for the treatment. However, in this study, there was no bias in the treatment line between patients with *EGFR* and *ALK* mutations and other cases. Third, this study did not include information on adverse effects. In melanoma patients, adverse effects, including vitiligo and rash, were reported to be good prognostic factors for patients treated with nivolumab [27,48]. 

## 4. Materials and Methods 

### 4.1. Patients

We retrospectively analyzed 190 advanced lung adenocarcinoma patients who received nivolumab or pembrolizumab (ICIs) monotherapy from January 2015 to September 2018 at the Aichi Cancer Center Hospital. The database was closed on 1 May 2019; at this time, 117 of the 190 patients had died. This study was approved by the Institutional Review Board in our institution (ACC-2019-1-002). The informed consent was waived because of the retrospective nature of this study.

Data on patient characteristics, genetic characteristics (*EGFR*, *KRAS*, *ALK*, *HER2*, and *BRAF*), baseline laboratory data (C-reactive protein (CRP), lactate dehydrogenase (LDH), neutrophil-to-lymphocyte ratio (NLR), and serum albumin level), PD-L1 expression of the tumors, objective response, PFS of ICI, and OS were obtained. Serum LDH and CRP levels were measured just before initiation of treatment with ICI. The cut-off values for LDH, CRP, albumin, and NLR were determined from standard values and previous reports [18,41,42,43] and in this study, we used the following levels: serum LDH: <245 vs. ≥245 IU/L, serum CRP: <1.0 vs. ≥1.0 mg/dL, serum albumin: <3.5 vs. ≥3.5 mg/dL, and NLR: <5 vs. ≥5.

### 4.2. Analysis of Efficacy of ICI

The patients received at least one infusion of nivolumab (3 mg/kg every 2 weeks) or pembrolizumab (200 mg/body every 3 weeks) monotherapy. The patients were treated with an ICI until they showed disease progression or experienced unacceptable adverse events. In general, the patients underwent radiographic imaging every 2 months and were evaluated for tumor response according to the Response Evaluation Criteria in Solid Tumors, version 1.1. The ORR was calculated as the total percentage of patients with a complete response or a partial response. 

### 4.3. Mutation Analyses of EGFR, ALK, KRAS, HER2, and BRAF

*EGFR* mutations (exons 18–21) were identified through the cycleave polymerase chain reaction (PCR) method. *HER2* (exon 20), *KRAS* (exons 2 and 3), and *BRAF* mutations (exons 11–15) were analyzed using fragment analysis, and the results were partially validated with direct sequencing, as previously reported [40]. *ALK* fusions were examined by reverse transcriptase (RT)-PCR, immunohistochemistry (IHC) analysis, or fluorescence in situ hybridization (FISH) assays (Vysis *ALK* Break Apart FISH Probe Kit; Vysis, Inc, Downers Grove, IL, USA). A tumor was considered to be *ALK* positive when two or more mutations were present or the RT-PCR, IHC, or FISH tests had positive results, as previously reported [49].

### 4.4. PD-L1 Expression Analysis

Tumor PD-L1 protein expression was evaluated during pre-treatment of (archival or recent) tumor biopsy or surgical resection specimens using an automated IHC assay. Depending on the drug, the antibody for companion diagnostic testing varied. The 28-8 pharmDx (Dako, North America, Carpinteria, CA, USA) and pembrolizumab use 22C3 pharmDx (Dako North America, Carpinteria, CA, USA). PD-L1 was evaluated, before initial treatment, with the 22C3 antibody for currently diagnosed cases. However, PD-L1 expression was evaluated by using the 28-8 antibody in cases treated with an ICI before pembrolizumab was approved. Therefore, two antibodies were used to examine some patients. In this study, PD-L1 expression was evaluated by using the values obtained by the antibodies used for companion diagnostic testing. The status of PD-L1 expression was measured as the proportion of PD-L1-expressing tumor cells in a section that included 100 or more tumor cells. In 190 patients, we identified 151 patients with tumor specimens that were evaluated for PD-L1 expression. 

### 4.5. Statistical Analysis

All statistical analyses were performed using the JMP version 11 statistical software package (SAS Institute, Cary, NC, USA). Differences in the baseline characteristics between the groups were compared by Fisher’s exact tests for categorical data. PFS was calculated from the date of therapy initiation to disease progression. OS was calculated from the date of nivolumab therapy initiation to death and censored at the date of the last visit for patients whose death could not be confirmed. The survival probabilities were estimated using the Kaplan–Meier method, where differences in the variables were calculated with the log-rank test. The multivariate regression analysis was performed according to the Cox proportional hazard model. Covariates with *P* ≤ 0.05 in the univariate analysis were included in the multivariate model. 

## 5. Conclusions

ICI treatment was significantly less efficacious in patients with *ALK* rearrangement than in patients with *EGFR* mutations, but there was no significant difference in efficacy between patients with other mutations and no mutations. ICIs have an important role in the treatment strategy for advanced NSCLC, but patients with *ALK* and *EGFR* mutations showed little benefit from ICIs. In addition, PD-L1 expression was not a critical biomarker for ICI treatment in patients with *EGFR* or *ALK* mutations. This is important information when choosing the next treatment after TKIs. Further investigation of the association of each driver oncogene with the efficacy of ICI treatment is warranted.

## Figures and Tables

**Table 1:** Patient characteristics.

Characteristics		N = 190
Age	Median (Range)	66 (32–87)
	<75 years	173 (91%)
	≤75 years	17 (9%)
Sex	Male	128 (67%)
	Female	62 (33%)
Stage	Relapse post-operation	32 (17%)
	III/IV	158 (83%)
ICI	Nivolumab	138 (73%)
	Pembrolizumab	52 (27%)
ECOG PS	0–1	166 (87%)
	2–3	24 (13%)
Histology	Adenocarcinoma	190 (100%)
Smoking	Smoker	141 (74%)
	Non-smoker	49 (26%)
Mutation	*EGFR*	47 (25%)
	*ALK*	7 (4%)
	Others	40 (21%)
	None	96 (51%)
PD-L1 status	Unknown	39 (21%)
	0	41 (22%)
	1%–49%	52 (27%)
	≥50%	58 (31%)

ICI, immune checkpoint inhibitor; ECOG: Eastern Cooperative Oncology Group; PS: Performance status; and PD-L1, program death-ligand 1.

**Table 2:** Univariate and multivariate analyses for progression-free survival of ICI-treated patients.

	Univariate				Multivariate
Objectives		Risk Ratio	95% CI	*p*-Value	
Sex	Male/Female	0.62	0.45–0.85	<0.01	0.37
Mutation	Without *EGFR*, *ALK/EGFR*, or *ALK*	0.54	0.39–0.75	<0.01	0.02
PS	0–1/2–3	0.24	0.15–0.38	<0.01	<0.01
PD-L1	Positive/negative	0.57	0.39–0.83	<0.01	0.01
Smoking	Smoker/non-smoker	0.59	0.42–0.83	<0.01	0.92
CRP	<1.0/≥1.0 mg/dL	0.65	0.48–0.88	<0.01	0.02
LDH	<245/≥245 U	0.59	0.43–0.81	<0.01	0.07
Albumin	<3.5/≥3.5 g/dL	0.68	0.48–0.98	0.04	0.8
NLR	<5.0/≥5.0	0.56	0.40–0.80	<0.01	0.33

PS, performance status; PD-L1, program death-ligand 1; CRP, C-reactive protein; LDH, lactate dehydrogenase; and NLR, neutrophil-to-lymphocyte ratio.

## Abbreviations

ALK	Anaplastic lymphoma kinase
EML4	Echinoderm microtubule-associated protein-like 4
NSCLC	Non-small-cell lung cancer
EGFR	Epidermal growth factor receptor
TKIs	Tyrosine kinase inhibitors
ICI	Immune checkpoint inhibitor
ABCP	Atezorizumab + bevacizumab + carboplatin + paclitaxel
PFS	Progression-free survival
OS	Overall survival
HR	Hazard ratio
CI	Confidence interval
PD-L1	Programmed death-ligand 1
KRAS	v-Ki-ras2 Kirsten rat sarcoma viral oncogene homolog
HER2	Human epidermal growth factor receptor type 2
BRAF	B-Raf proto-oncogene, serine/threonine kinase
ORR	Objective response rate
TIL	Tumor-infiltrating lymphocytes
CRP	C-reactive protein
LDH	Lactate dehydrogenase
NLR	Neutrophil-to-lymphocyte ratio
PCR	Reverse transcriptase
IHC	Immunohistochemistry
FISH	Fluorescence in situ hybridization
